# Mixed protocols: Multiple ratios of FSH and LH bioactivity using highly purified, human-derived FSH (BRAVELLE) and highly purified hMG (MENOPUR) are unaltered by mixing together in the same syringe

**DOI:** 10.1186/1477-7827-3-61

**Published:** 2005-11-09

**Authors:** M Joseph Scobey, Elizabeth Raike, Dennis C Marshall

**Affiliations:** 1 Ferring Pharmaceuticals Inc., Suffern, New York, USA; 2 Qualtech Laboratories Inc., Ocean, New Jersey, USA

## Abstract

**Background:**

The use of mixed or blended protocols, that utilize both FSH and hMG, for controlled ovarian hyperstimulation is increasing in use. To reduce the number of injections a patient must administer, many physicians instruct their patients to mix their FSH and hMG together to be given as a single injection. Therefore, the goal of this study was to definitively determine if the FSH and LH bioactivities of highly purified, human-derived FSH (Bravelle(R)) and highly purified hMG (Menopur(R)) were altered by reconstituting in 0.9% saline and mixing in the same syringe.

**Methods:**

Bravelle(R) and Menopur(R) were reconstituted in 0.9% saline and mixed in a Becton Dickinson plastic syringe. The FSH and LH bioactivities of the products were determined after injecting female and male rats, respectively, with Bravelle(R), Menopur(R), or a mixture of Bravelle(R) and Menopur(R). Ratios of FSH:LH activity tested were 150:75 IU (1 vial Bravelle(R): 1 vial Menopur(R)), 300:75 IU (3 vials Bravelle(R): 1 vial Menopur(R)) or 300:225 IU (1 vial Bravelle(R): 3 vials of Menopur(R)).

**Results:**

There were no statistically significant changes in either FSH or LH bioactivity that occurred after mixing Bravelle(R) with Menopur(R) in the same syringe. The theoretical vs. actual FSH bioactivity for Bravelle(R) and Menopur(R) were 75 vs. 76.58 IU/mL and 75 vs. 76.0 IU/mL, respectively. For the 3 ratios of FSH:LH activity tested, 150:75 IU (1 vial Bravelle(R): 1 vial Menopur(R)), 300:75 IU (3 vials Bravelle(R): 1 vial Menopur(R)) or 300:225 IU (1 vial Bravelle(R): 3 vials of Menopur(R)) tested, the theoretical vs. actual FSH bioactivities were 150 vs. 156.86 IU/mL, 300 vs. 308.69 IU/mL and 300 vs. 306.58 IU/mL, respectively. The theoretical vs. actual LH bioactivity for Menopur(R) in the above mentioned ratios tested were 75 vs. 77.50 IU/mL. For the 3 ratios of FSH:LH activity tested, 150:75 IU (1 vial Bravelle(R): 1 vial Menopur(R)), 300:75 IU (3 vials Bravelle(R): 1 vial Menopur(R)) or 300:225 IU (1 vial Bravelle(R): 3 vials of Menopur(R)), the theoretical vs. actual LH bioactivities were 75 vs. 78.38 IU/mL, 75 vs. 78.63 IU/mL and 225 vs. 233.48 IU/mL, respectively.

**Conclusion:**

Mixing human-derived FSH (Bravelle(R)) with highly purified hMG (Menopur(R)) in the same diluent, 0.9% NaCL, does not alter the FSH or LH bioactivity of either gonadotropin preparation.

## Introduction

Greep and co-workers [1] were the first to demonstrate that follicle stimulating hormone (FSH) and luteinizing hormone (LH) were distinct chemical moieties and that both were necessary for follicular growth, ovulation and formation of the corpus luteum. Sixteen years later, pituitary-derived gonadotropin extracts were used successfully to induce ovulation in anovulatory women [2]. Since then, gonadotropins have been widely used to successfully treat infertile women with oligomenorrhea and amenorrhea that is not secondary to ovarian failure [3].

The first commercially available gonadotropin, human menopausal gonadotropin (hMG; Pergonal^®^), was purified from the urine of postmenopausal women and contained approximately equal amounts of FSH and LH activity. By adsorbing LH using anti-hCG antibodies and filtration through gel chromatography columns, an FSH only preparation was subsequently produced from postmenopausal urine [4]. In 1986, urinary FSH with <1% LH activity became available for clinical use. A preparation devoid of LH was speculated to have a theoretical advantage over hMG in controlled ovarian hyperstimulation (COH) protocols in women with polycystic ovary syndrome (PCOS) since these women have elevated levels of endogenous LH [5]. However, to date there is no convincing evidence to support that in PCOS patients, FSH alone is more effective than hMG for COH.

Today, COH protocols that utilize both hMG and FSH, known as mixed or blended protocols, are commonly used in an attempt to obtain higher pregnancy rates. To reduce the number of daily injections, physicians often recommend that patients reconstitute the different hormone preparations in the accompanying diluent and then mix them in the same syringe. This practice raises the question of gonadotropin compatibility, especially if products from different manufacturers and/or different diluents are used. Although amino acid sequences of human and recombinant derived FSH are identical, they differ with respect to their glycosylation profile [6]. This can affect the chemical nature of these glycoproteins and impact their interaction with each other in a heterogeneous mixture.

In a recent study [7], it was demonstrated that highly purified, human-derived FSH (Bravelle^®^; Ferring Pharmaceuticals Inc., Suffern, New York) could be reconstituted in 0.9% saline and mixed with hMG (Repronex^®^; Ferring Pharmaceuticals Inc., Suffern, New York) in the same syringe, without any alteration of the FSH and LH bioactivities of either product. The objective of the present investigation was to determine if the FSH and LH bioactivities of highly purified, human-derived FSH (Bravelle^®^) and a new, highly purified hMG (Menopur^®^; Ferring Pharmaceuticals Inc., Suffern, New York) were altered when reconstituted in 0.9% saline and mixed in the same syringe.

## Materials and methods

### Study Design

Validated methods [8], specified by the United States Pharmacopeia (USP), were used to determine the bioactivities of each product individually and subsequent mixtures of the two. The bioassays were performed at Qualtech Laboratories Inc., Ocean, New Jersey. Ovarian weights (FSH bioassay) or seminal vesicle weights (LH bioassay) were obtained after treating prepubertal female or male rats with Bravelle^® ^(75 IU FSH; ≤2% LH activity), Menopur^® ^(75 IU FSH; 75 IU LH activity), or one of three mixtures of Bravelle^® ^and Menopur^®^: 150:75 IU (1 vial Bravelle^®^:1 vial Menopur^®^); 300:75 IU (3 vials Bravelle^®^: 1 vial Menopur^®^); and 300:225 IU (1 vial of Bravelle^®^:3 vials of Menopur^®^). These ratios represent a low combination of FSH: LH activity (150:75 IU) as well as combinations with a high FSH and low LH activity (300:75 IU) and high FSH and high LH activity (300:225 IU). The FSH and LH bioactivities were compared to a menotropin Reference Standard traceable to the National Institute for Biological Standards and Control (NIBSC).

### Test Animals

In-bred Wistar female rats (n = 296) 21 days of age were used for the FSH bioassay and Sprague Dawley male rats (n = 248) 21 days of age were used for the LH bioassay. The rats, certified to be free from murine viruses, were purchased from Hilltop Lab Animals Inc. (Scottdale, Pennsylvania). Upon arrival, the animals were weighed, their health was assessed and they were randomized into groups of eight. The animals were housed in polypropylene solid bottom cages with stainless steel wire lids containing shredded Aspen shavings. The animal room was maintained at 18–19°C with a relative humidity of approximately 50% and 12:12 hour, light: dark cycle. Rats were given Purina rodent diet and tap water *ad libitum*.

For both the FSH and LH assays, one group of rats (N = 8) was assigned to each of the low, middle, and high concentration groups for each gonadotropin, gonadotropin mixture and Reference Standard. Each assay was done in duplicate, Therefore, for the FSH assay, 48 rats each were assigned to six groups: Bravelle^®^, Menopur^®^, the three ratios of Bravelle^® ^plus Menopur^®^, and the Reference Standard. For the LH assay, 48 rats each were assigned to five groups: Menopur^®^, the three ratios of Bravelle^® ^plus Menopur^®^, and the Reference Standard. In addition, there was a control group in each assay that had eight rats treated with diluent alone.

### Reference Standards for the FSH and LH assays

The Reference Standard diluent used for both gonadotropins was prepared by dissolving 10.75 g disodium hydrogen phosphate, 7.6 g sodium chloride and 1.0 g bovine serum albumin in 1.0 liter of distilled water. The pH of the solution was adjusted to 7.2 ± 0.2 with 1 N sodium hydroxide.

As per the USP, for the FSH assay 70,000 units of hCG were added to the Reference Standard diluent. A menotropin Reference Standard (Ferring, lot-DPH12250397, 122.2 FSH IU/mg, traceable to NIBSC) was reconstituted and diluted in the Reference Standard diluent to the final concentrations of 1.9, 3.8 and 7.6 IU/0.6 mL.

For the LH assay, a menotropin Reference Standard (Ferring, lot-DPH12250397, 99.3 LH IU/mg, traceable to NIBSC) was reconstituted and diluted in the Reference Standard diluent to the final concentrations of 7, 14, and 28 IU/0.8 mL.

The Reference Standard concentrations were established in a geometric progression for the low, middle and high doses respectively, based on previous dose response studies. The lowest concentration in this three-dose range produces a definite response in some of the rats as compared to the control group and the highest concentration produces a submaximal to maximal response.

#### Gonadotropin Assays

##### Bravelle^® ^and Menopur^® ^single solutions

For the FSH assay, four vials of Bravelle^® ^(lot-FMA001) or Menopur^® ^(lot-FHA004ULB) were each individually reconstituted with 1.0 mL of 0.9% saline and pooled. Each solution was adjusted with Reference Standard diluent to the same three concentrations as the reference standard (1.9, 3.8 and 7.6 IU/0.6 mL). For the LH assay, fourteen vials of Menopur^® ^were each individually reconstituted with 1.0 mL of 0.9% saline and pooled. The solution was adjusted with Reference Standard diluent to the same three concentrations as the reference standard (7, 14 and 28 IU/0.8 mL).

##### Analyte (Mixture of Bravelle^® ^and Menopur^®^)

###### 150:75 IU; FSH: LH

For both the FSH and LH bioassay 20 vials of Bravelle^® ^(lot-FMA001) were each individually reconstituted with 1.0 mL of 0.9% saline and pooled. For the FSH assay, two vials of Menopur^® ^(lot-FHA004ULB) were each individually reconstituted with 1.0 mL of the Bravelle mixture and pooled. For the LH assay analyte, 14 vials of Menopur^® ^(lot-464-891) were each individually reconstituted with 1.0 mL of the Bravelle^® ^mixture and pooled.

###### 300:75 IU; FSH: LH

For both the FSH and LH bioassay 56 vials of Bravelle^® ^(lot-FMA001) were each individually reconstituted with 0.5 mL of 0.9% saline and pooled (diluent A). Another 20 vials of Bravelle^® ^(lot-FMA001) were each individually reconstituted with 1.0 mL of diluent A and pooled (diluent B). For the FSH assay, two vials of Menopur^® ^(lot-FHA004ULB) were each individually reconstituted with 1.0 mL of diluent B and pooled. For the LH assay analyte, 14 vials of Menopur^® ^(lot-FHA004ULB) were each reconstituted with 1.0 mL of diluent B and pooled.

###### 300:225 IU; FSH: LH

For both the FSH and LH bioassay, 29 vials of Menopur^® ^(lot-FHA004ULB) were each individually reconstituted with 0.5 mL of 0.9% saline and pooled (diluent A). Another 10 vials of Menopur^® ^(lot-FHA004ULB) were individually reconstituted with 1.0 mL of diluent A and pooled (diluent B). For the FSH assay, two vials of Bravelle^® ^(lot-FMA001) were each reconstituted with 1.0 mL of diluent B and pooled. For the LH assay, five vials of Bravelle^® ^(lot-FMA001) were each individually reconstituted with 1.0 mL of diluent B and pooled.

For all mixtures, reconstitution and pooling were done with commercially available BD plastic syringes. The solutions were further diluted to the same three concentrations as the Reference Standard with Reference Standard diluent: 1.9; 3.8; and 7.6 IU/0.6 mL for the FSH assay and 7; 14; and 28 IU/0.8 mL for the LH assay.

##### Control Solution

The Reference Standard diluent was used as the control solution for both assays.

##### FSH assay

Each female rat was injected subcutaneously with 0.2 mL of the assigned dose at approximately the same time of day for three consecutive days. Twenty-four hours after the last injection, rats were sacrificed in a carbon dioxide chamber. Left and right ovaries were carefully dissected from each rat, freed of fat or fibrous tissue, dried by gentle blotting on absorbent paper and weighed on an analytical balance. For each rat, the left and right ovarian weights were recorded.

##### LH assay

Each male rat was injected with 0.2 mL of the assigned dose at approximately the same time of day for four consecutive days. Twenty-four hours after the last injection, rats were sacrificed in a carbon dioxide chamber. Seminal vesicles were carefully dissected from each rat, freed of fat or fibrous tissue, dried by gentle blotting on absorbent paper and weighed on an analytical balance.

### Determination of FSH and LH bioactivity: potency calculations

The FSH and LH assays were 3 × 3 parallel line assays performed in duplicate. For each assay, the response to three concentrations of the test hormones and the analyte were compared to the response of the same three concentrations of the Reference Standard. The result from each replicate was combined for the final result. For each replicate, ovarian weights or seminal vesicle weights were used to calculate potency values for FSH and LH, respectively.

For potency values to be accepted, the combined L-value (the confidence limit) from the duplicate assays had to be less than 0.08 (L-value set by Ferring Pharmaceuticals Inc.) which means that the true bioactivity of the preparation was within 93–107% of the obtained result. By Ferring imposed standards, in order to have accurate, consistent assesments of bioactivity, if an L-value was not met, the bioassay was to be repeated until the combined L-value was less than 0.08, providing a more exacting bioassay. This is a more stringent criterion than specified in the USP monograph on menotropins, which only requires an L-value of 0.18 (a value ±21% of the obtained result) [8].

Potency calculations were the same for the FSH and LH bioassays. The following equations were used: M' = ciT_a_/T_b_; where M' = log-potency of an unknown relative to its assumed potency; c = 4/3; i = interval in logarithms between successive log-doses (same for standard and test concentration); T_a _= difference in responses between the standards and the test concentrations, T_b _= combined slope of the dose-response curves for the standards and test concentrations. After calculating M', the log-potency was determined by the equation: M = M'+log R, (M = log potency; for the present experiment: R = 1 making log R = 0). Therefore, potency = antilog M, and % claim = antilog M × 100 [8]. FSH and LH bioactivities obtained by these assays were compared with the FSH and LH bioactivities set forth in the product label (labeled claim).

### Statistical analysis

An analysis of variance (ANOVA) was performed to determine if there were differences among ovarian or seminal vesicle weights between replicates. This analysis included: replicate; treatment; dose; replicate × treatment; replicate × dose; and replicate × treatment × dose, as sources of variation. Since replicate × treatment × dose analyses were not significant (P > 0.05), ovarian weights from both replicates were combined as were seminal vesicle weights for both replicates.

An ANOVA for a randomized block design was next performed to determine if there were differences in ovarian or seminal vesicle weights among treatments. This analysis included: replicate (block); treatment; dose and treatment × dose, as sources of variation.

## Results

### FSH Bioassay

**Figure 1:**
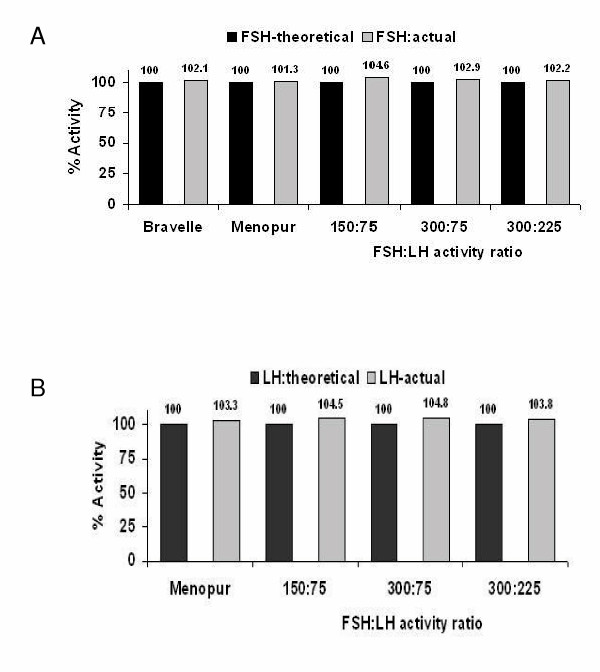
**Theoretical vs. actual FSH and LH activities**. A) FSH bioactivities of Bravelle, Menopur and the three ratios of Bravelle and Menopur expressed as a percent of labeled claim. B) LH bioactivities of Menopur and the three ratios of Bravelle and Menopur expressed as a percent of labeled claim.

The ovarian weights (mean ± SEM) were not significantly different among treatments (Table 1). As expected, ovarian weights increased in a dose-dependent manner with increasing doses of FSH. The magnitude of increase in ovarian weights was similar across treatment groups (treatment × dose, P > 0.05; Table 1), indicating that FSH bioactivity was unaffected by either reconstitution of hormones with 0.9% saline or by mixing the reconstituted hormones in the same syringe. The theoretical and actual FSH bioactivities for Bravelle^®^, Menopur^® ^and a mixture of Bravelle^® ^and Menopur^® ^are shown in Table 2. The bioactivities of the five groups expressed as a percent of the labeled claims are shown in Figure 1a.

**Table 1:** Ovarian weights in rats injected with a low, middle and a high dose of the Reference Standard, Bravelle^®^, Menopur^®^, or a mixture of Bravelle^® ^and Menopur^® ^reconstituted in 0.9% saline and mixed in the same syringe.

Hormone (FSH:LH)^a^	Total no. rats	Ovarian weights (mean ± SEM)^b^*		
		
		Low^c ^dose	Middle^d ^dose	High^e ^dose
Reference Standard	48	93.55 ± 4.17	151.37 ± 4.00	193.01 ± 5.02
Bravelle^® ^(75:0)	48	92.31 ± 3.02	149.60 ± 3.29	201.37 ± 4.56
Menopur^® ^(75:75)	48	92.19 ± 3.45	151.51 ± 2.62	194.92 ± 4.03
Bravelle^® ^+ Menopur^®^				
(150:75)	48	97.29 ± 2.80	148.82 ± 3.65	199.59 ± 4.30
(300:75)	48	92.38 ± 3.37	157.08 ± 3.82	192.62 ± 5.50
(300:225)	48	95.75 ± 2.94	149.50 ± 6.11	195.39 ± 6.13

* The magnitude of this increase was similar across treatment groups (treatment × dose P = 0.71)

^a ^Ratio of FSH:LH bioactivity expressed in IUs

^b ^Combined ovarian weights from the 2 replicates (replicate × treatment × dose)

^c ^Low dose = 1.9 IU FSH/0.6 mL

^d ^Middle dose = 3.8 IU FSH/0.6 mL

^e ^High dose = 7.6 IU FSH/0.6 mL

**Table 2:** Theoretical and actual FSH bioactivities for Bravelle^®^, Menopur^®^, and a mixture of Bravelle^® ^and Menopur^® ^when reconstituted in 0.9% saline and mixed in the same syringe.

Hormone (FSH:LH)^a^	Theoretical bioactivity IU/mL (%)	Actual bioactivity IU/mL (% claim)	L-value^b^
Bravelle^® ^(75:0)	75 (100%)	76.58 (102.1%)	0.072
Menopur^® ^(75:75)	75 (100%)	76.0 (101.3%)	0.072
Bravelle^® ^+ Menopur^®^			
(150:75)	150 (100%)	156.86 (104.6%)	0.072
(300:75)	300 (100%)	308.69 (102.9%)	0.072
(300:225)	300 (100%)	306.58 (102.2%)	0.072

^a ^Ratio of FSH:LH bioactivity expressed in IUs

^b ^L-value refers to the confidence limit obtained after combining two replicates. The USP mandated L-value = 0.18; the more stringent L-value of 0.08 is set by Ferring Pharmaceuticals Inc. to more precisely and consistently assess bioactivity.

### LH Bioassay

The seminal vesicle weights (mean ± SEM) were not significantly different among treatments (Table 3). As expected, seminal vesicle weights increased with increasing doses of LH (Table 3). The magnitude of this increase was similar across treatment groups (treatment × dose, P > 0.05; Table 3), indicating that LH bioactivity was unaffected by either reconstitution of hormones with 0.9% saline or by mixing the hormones in the same syringe. The theoretical and actual bioactivities for Menopur^® ^and a mixture of Bravelle^® ^and Menopur^® ^are shown in Table 4. The bioactivities of the four groups expressed as a percent of the labeled claims are shown in Figure 1b.

**Table 3:** Seminal vesicle weights in rats injected with a low, middle and a high dose of the Reference Standard, Menopur^®^, or a mixture of Bravelle^® ^and Menopur^® ^reconstituted in 0.9% saline and mixed in the same syringe.

Hormone (FSH:LH)^a^	Total no. rats	Seminal Vesicle weights (mean ± SEM)^b^*		
		
		Low^c ^dose	Middle^d ^dose	High^e ^dose
Reference Standard	48	35.82 ± 1.64	68.88 ± 2.40	96.39 ± 2.95
Menopur^® ^(75:75)	48	36.49 ± 1.54	72.55 ± 1.61	95.69 ± 2.87
Bravelle^®^+ Menopur^®^				
(150:75)	48	36.47 ± 1.23	73.94 ± 2.42	96.62 ± 1.70
(300:75)	48	37.80 ± 1.25	74.57 ± 1.57	94.49 ± 2.09
(300:225)	48	36.28 ± 1.50	74.78 ± 1.78	95.73 ± 2.01

* The magnitude of this increase was similar across treatment groups (treatment × dose P = 0.74)

^a ^Ratio of FSH:LH bioactivity expressed in IUs

^b ^Combined seminal vesicle weights from the 2 replicates (replicate × treatment × dose)

^c ^Low dose = 7 IU LH/0.8 mL

^d ^Middle dose = 14 IU LH/0.8 mL

^e ^High dose = 28 IU LH/0.8 mL

**Table 4:** Theoretical and actual LH bioactivities for Menopur^®^, and a mixture of Bravelle^® ^and Menopur^® ^when reconstituted in 0.9% saline and mixed in the same syringe.

Hormone (FSH:LH)^a^	Theoretical bioactivity IU/mL (%)	Actual bioactivity IU/mL (% claim)	L-value^b^
Menopur^® ^(75:75)	75 (100%)	77.50 (103.3%)	0.061
Bravelle^® ^+ Menopur^®^			
(150:75)	75 (100%)	78.38 (104.5%)	0.061
(300:75)	75 (100%)	78.63 (104.8%)	0.061
(300:225)	225 (100%)	233.48 (103.8%)	0.061

^a ^Ratio of FSH:LH bioactivity expressed in IUs

^b ^L-value refers to the confidence limit obtained after combining two replicates. The USP mandated L-value = 0.18; the more stringent L-value of 0.08 is set by Ferring Pharmaceuticals Inc. to more precisely and consistently assess bioactivity.

While Bravelle^® ^contains up to 2% LH, due to the small amount, for this assay it is assumed that Bravelle^® ^does not contain LH activity.

For both the FSH and LH bioassays, the L-value obtained was less than 0.08 which means that the true bioactivity is within 93–107% of the obtained result. Since all of the required USP specifications for the bioassays were met, it is concluded that the resulting bioactivity for LH and FSH in the mixed preparations was unaffected by reconstituting Bravelle^® ^and Menopur^® ^in 0.9% saline and mixing in a BD plastic syringe.

## Discussion

In many cases, physicians are administering FSH and hMG as separate injections because of concerns that different gonadotropins may not be compatible, which could result in unexpected results during controlled ovarian hyperstimulaiton (COH) cycles. This concern for compatibility has merit since different gonadotropins are either from animal or human origin and use different diluents for reconstitution and/or require different delivery systems. It is well known that even structurally similar proteins, expecially those of high molecular weight can be incompatible. FSH, LH and hCG gonadotropins have high molecular weight (27–31 kD) and require surfactants to prevent sticking. Nonetheless, in an effort to minimize the number of injections that a woman must take while undergoing COH, some physicians are combining FSH and hMG in the same syringe. The charge distribution (described by the isoelectric point) and the carbohydrate complexity (simple, intermediate and complex carbohydrates) of recombinant FSH was found to be quite different when compared with serum FSH throughout the menstrual cycle [11]. Therefore, when hormones with different chemical configurations are reconstituted in different diluents and mixed, their compatibility and possibly their bioactivity may be affected.

A previous study demonstrated that the activity of peptides may be significantly changed by the diluents in which they are dissolved [12] or the characteristic of the vessel used for mixing. Studies have shown that peptides and proteins adhere to certain surfaces [13]. Surface adsorption of calcitonin on soda lime silica glass is pH dependent [13]. Fibrinogen adheres to both dimethyldichlorosilane-treated glass and low-density polyethylene [14].

We must emphasize that the results from this study and a previously published study [7] were obtained with FSH and LH activity containing preparations from a single manufacturer (Ferring Pharmaceuticals Inc.) and that the hormone preparations were reconstituted and mixed according to the manufacturer's directions. These data cannot be extended to other preparations of gonadotropins, particularly if recombinant hormones that require bacteriostatic water for reconstitution or other delivery vehicles are mixed with human-derived hormone preparations.

In summary, the results of this study confirm those of a previous study [7] combining Bravelle^® ^with Repronex^®^. The present study was conducted because Menopur is a more highly-purified hMG than Repronex^®^, thereby resulting in significantly fewer injection site reactions [15]. Since Menopur is now commercially available for use in ART, it is important for doctors to have confidence that Bravelle^® ^and Menopur^® ^can be reconstituted with 0.9% saline (diluent provided by the manufacturer) and mixed together in a commercially available syringe without altering the FSH or LH bioactivity. The health care provider can be assured that the expected doses of FSH and LH will be delivered, with no alteration in bioactivity of either Bravelle^® ^or Menopur^®^.
